# Comparative and evolutionary analyses of the divergence of plant oligosaccharyltransferase STT3 isoforms

**DOI:** 10.1002/2211-5463.12804

**Published:** 2020-02-19

**Authors:** Guanting Niu, Zhuqing Shao, Chuanfa Liu, Tianshu Chen, Qingsong Jiao, Zhi Hong

**Affiliations:** ^1^ State Key Laboratory of Pharmaceutical Biotechnology NJU Advanced Institute for Life Sciences (NAILS) School of Life Sciences Nanjing University China; ^2^ Department of Biology Institute of Plant and Food Science Southern University of Science and Technology Shenzhen China

**Keywords:** evolution, N‐glycosylation, oligosaccharyltransferase, selection pressure, subunit cooperation

## Abstract

STT3 is a catalytic subunit of hetero‐oligomeric oligosaccharyltransferase (OST), which is important for asparagine‐linked glycosylation. In mammals and plants, OSTs with different STT3 isoforms exhibit distinct levels of enzymatic efficiency or different responses to stressors. Although two different STT3 isoforms have been identified in both plants and animals, it remains unclear whether these isoforms result from gene duplication in an ancestral eukaryote. Furthermore, the molecular mechanisms underlying the functional divergences between the two STT3 isoforms in plant have not been well elucidated. Here, we conducted phylogenetic analysis of the major evolutionary node species and suggested that gene duplications of STT3 may have occurred independently in animals and plants. Across land plants, the exon–intron structure differed between the two STT3 isoforms, but was highly conserved for each isoform. Most angiosperm STT3a genes had 23 exons with intron phase 0, while STT3b genes had 6 exons with intron phase 2. Characteristic motifs (motif 18 and 19) of STT3s were mapped to different structure domains in the plant STT3 proteins. These two motifs overlap with regions of high nonsynonymous‐to‐synonymous substitution rates, suggesting the regions may be related to functional difference between STT3a and STT3b. In addition, promoter elements and gene expression profiles were different between the two isoforms, indicating expression pattern divergence of the two genes. Collectively, the identified differences may result in the functional divergence of plant STT3s.

## Graphical abstract

STT3 is a catalytic subunit of oligosaccharyltransferase (OST) which is important for N‐glycosylation. Two subtypes of STT3 exist in animal and plant through independent duplications. An ancient separation of STT3 in the common ancestor of plants was discovered. Different elements in the promoters, distinct motifs, and auxiliary subunits may confer divergent catalytic ability on OST containing STT3a or STT3b.

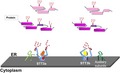

**Abbreviations**
ER: endoplasmic reticulum
GlcNAc: N‐acetylglucosamine
Man: mannose
N‐glycosylation: asparagine‐linked glycosylation
OST: oligosaccharyltransferase

## Introduction

Plant and animal development differs radically, and yet, many posttranslational modifications are conserved across both groups 1. Asparagine‐linked glycosylation (N‐glycosylation) is one of the most significant and abundant posttranslational protein modifications. This process has been documented in the three domains of life and is involved in various biological processes 2, 3. Overall, > 50% of all proteins, across all three domains, may be modified by sugar molecules during their life cycle 4, 5. Oligosaccharides are important secondary metabolites in plants, which play a significant role in detoxification 6. Oligosaccharides also regulate plant growth homeostasis, in conjunction with auxins, gibberellins, and brassinolides 7, 8, 9, 10. In many proteins, including EF‐TU receptor, a well‐characterized leucine‐rich repeat receptor kinases, which folding processes, stability, and even function are influenced by N‐glycosylation defects 11, 12, 13.

N‐glycosylation trimming is a process that is conserved across eukaryotes. In this process, the lipid‐linked oligosaccharide is assembled on a lipid anchor and turned from the cytosolic to the luminal side of the eukaryotic endoplasmic reticulum (ER) membrane. Subsequently, monosaccharides are incorporated stepwise by a series of glycosyltransferases (GTs) to form a tetradecasaccharide (Glc_3_Man_9_GlcNAc_2_) 14. GTs are particularly important because glycan moiety forms are conferred to lipids and nascent peptides to form essential components of natural products; these products have various biological properties, such as molecule transportation, toxicity reduction, stabilization, and solubility enhancement 15. As of August 2010, 456 GT genes had been identified in *Arabidopsis thaliana*, 226 in *Homo sapiens*, and 149 in *Drosophila melanogaster.* By 2015, these GTs were classified into 97 families (GT1–GT97) (http://www.cazy.org/GlycosylTransferases) 16, 17. Secretory proteins are synthesized in the rough ER and modified on the lumen side of the membrane by a catalytic subunit (STT3) of the oligosaccharyltransferase (OST) affiliated with GT66. When translocated to the ER lumen, STT3 transfers the Glc_3_Man_9_GlcNAc_2_‐pp‐dolichol en bloc to the asparagine at the N‐X‐S/T (N: asparagine, X ≠ Proline, S: serine, T: threonine) sites within the nascent polypeptides and assists them to their final intra‐ or extracellular locations 18. After the protein is correctly folded, three glucose residues are removed and the glycoprotein exported to the Golgi apparatus for further glycan processing. *Saccharomyces cerevisiae* OST, the most incisive model in eukaryotes, includes eight different subunits: Ost1p, Ost2p, Ost4p, Ost5p, OST3p/Ost6p, Stt3p, Swp1p, and Wbp1p. Of these, five are essential for cell viability 19. Two mammalian OST complexes are composed of one copy of a subunit (STT3A or STT3B) and a shared set of noncatalytic subunits including isoform‐specific subunits 20. In plant, general appearances of OST–ribosome complex containing STT3a were visualized by transmission electron microscopy (TEM) and single particle analysis although the subunit arrangement is not clear 21. In this multimeric protein, STT3, the most conserved subunit, acts as a catalyst, while the auxiliary subunits fine‐tune the glycosylation process. For instance, Ost3/6p exhibits oxidoreductase activity and assists in the binding of specific polypeptides via both noncovalent and transient disulfide bonds 5. Cross‐linking analysis showed that mammalian RPN1 (Ost1p homologue) chaperones selected protein clients to the OST complex and presented them to the catalytic core 22. In plant, OST3/6 interacts with STT3a and OST4; in addition, it affects innate immunity and tolerance to abiotic stresses by N‐glycosylation deficiency 23. Defective glycosylation‐1 plays a role in cell growth and differentiation in plants 24. STT3, which is the catalytic subunit, contains an active center formed by the WWDYG and DXXK motifs 25, 26. STT3s are divided into two subtypes in animals and plants: STT3a and STT3b. The STT3 orthologues archaeal glycosylation B (AglB) and PglB alone account for all OST activity in archaea and bacteria 27, 28. Three paralogous genes *TbSTT3a*, *TbSTT3b*, and *TbSTT3c*, which encode the single subunit enzymes (STT3 homologue), discriminate biantennary and triantennary sugars, and control the oligosaccharide chains transfer of acidic and neutral regions of the polypeptide in *Trypanosoma brucei* 29. Mammalian OST isoforms STT3A and STT3B in the canine pancreas act on the flexible portions of the co‐ and postprotein modifications, respectively, and have different effects on the C‐terminal glycosylation sites 30. In humans, homozygous mutations in either STT3A or STT3B result in neurologic abnormalities, intellectual disabilities, and failure to thrive 31. In plants, two STT3 isoforms were identified in the *A. thaliana* genome. The *stt3a* mutant was sensitive to salt and pathogens, while the *stt3b* mutant was not. In addition, a double mutation in both *stt3a* and *stt3b* is lethal at the gamete stage, which suggests that these isoforms have both divergent and redundant functions 32. Although two different STT3 isoforms have been identified in both plants and animals, it remains unclear whether these isoforms result from gene duplication in an ancestral eukaryote. Furthermore, the molecular mechanisms underlying the functional divergences between the two STT3 isoforms in plant have not been well elucidated.

To investigate the evolution and divergence of the STT3 genes in eukaryotes, particularly plants, we constructed a phylogeny of STT3 genes from representative eukaryotic genomes, including animals, plants, and fungi. Our data suggested that independent gene duplications have led to the divergence of STT3 isoforms in animals and plants. The separation of the two STT3 clades in plants was traced to the common ancestor of green plants. The two STT3 clades are highly conserved in land plants, with clade‐specific gene structures and protein motifs. Clade‐specific differences in the cis elements of the promoter region, as well as gene expression patterns, also indicated that the isoforms encoded by the two STT3 clades were functionally divergent. Motifs specific to each STT3 were identified. Finally, selection pressure analyses showed that the amino acid regions under lower evolutionary constraint were identical to those regions containing motifs specific to STT3a and STT3b. Overall, our results suggested that genetic differences and specific motifs may underlie the functional differences between STT3a and STT3b.

## Materials and methods

### Retrieval of STT3 homologous sequences

Selected plant, animal, and fungus sequences were downloaded from JGI PHYTOZOME v12 (https://phytozome.jgi.doe.gov/pz/portal.html), ENSEMBL (http://ftp.ensembl.org/), fungal genome databases (http://fungalgenomes.org/data/), Saccharomyces Genome Database (http://www.yeastgenome.org), and other databases. Protein sequence queries were used to search for homologue by BLASTP with an *E* value of < 1 × 10^–5^. Pfam database was used to identify all proteins containing a STT3 domain (PF02516). In proteome datasets, if two or more protein sequences at the same locus were identical where they overlapped, we selected the longest sequence. The species used in this analysis contains a four‐letter species designation from the first letter of the genus and the first three letters of the species. Additional lowercase suffix indicated by gene locus number.

### Sequence alignment and phylogenetic analysis

The coding sequence (CDS) of all obtained STT3 genes were aligned using the ClustalW program that integrated in mega 5.0 33 with the default parameters. The resulted alignment was used for subsequent phylogenetic analysis. The phylogenetic analysis was performed by the seaview (Université de Lyon, Lyon, France) 34 software using the Maximum‐likelihood (ML) method with a bootstrap test of 1000 replicates.

The amino acid sequence of OST1 was aligned using the ClustalW program that integrated in mega 5.0 33 with the default parameters. The obtained alignment was subjected to the seaview 34 software for phylogenetic analysis using the ML method and with a bootstrap test of 1000 replicates.

The resulting trees were visualized and adjusted by figtree 1.3.1 (Ashworth Laboratories, Edinburgh, UK) (http://tree.bio.ed.ac.uk/software/figtree/).

### Gene structure and amino acid motif analysis

The intron position and phase for STT3s were determined by align the full‐length gene sequences and coding DNA sequences (CDS) for different species. Intron maps were constructed by determining the intron splice site phase and position. The following three intron phases were marked depending on their position relative to the reading frame: phase 0 (intron insertion between two codons), phase 1 (insertion after the first base of a codon), or phase 2 (insertion after the second base of a codon).

An 800‐bp genomic region upstream of the translation start site (ATG) was extracted for each STT3 gene to evaluate the presence of cis‐regulatory elements in the promoter regions, using PlantCARE database 35. The protein sequences of the STT3 homologues were analyzed by MEME website (http://meme-suite.org/tools/meme) to detect conserved motifs. We use classic mode and confine 40 motifs to be found with zero or one motif sites occurrence per sequence.

### Sliding window *K*_a_/*K*_s_ analysis

The ratio of the number of nonsynonymous substitutions per nonsynonymous site (*K*_a_) to the number of synonymous substitutions per synonymous site (*K*_s_), termed *K*_a_/*K*_s_ or dN/dS, was analyzed by the DNAsp software (http://www.ub.edu/dnasp/) using the alignment of the CDS sequences of STT3 genes. The sliding window and the step size were set to 50 and 10 bp, respectively.

### Gene expression data analysis

The expression of STT3 genes from different plant species was evaluated by Genevestigator (https://genevestigator.com/gv/). GENEVESTIGATOR is a high‐performance database and analysis tool for gene expression. It integrates thousands of manually curated, well‐described public microarray and RNA‐Seq experiments and nicely visualizes gene expression across different biological contexts. It contains expression data for *Arabidopsis* and some other plants of 134 different experimental conditions, tissues, and developmental stages. Expression levels and tissue‐specific expression of STT3 genes were visualized using the heatmap package integrated in Genevestigator.

### 3D structure analysis of the STT3 homologue

The 3D structures of STT3 homologue proteins were generated using the amino acid sequences. For this purpose, Swiss‐Model (https://swissmodel.expasy.org/) was used in an automated mode. The hidden Markov model‐based hmmer program (2.3.2) (https://www.ebi.ac.uk/Tools/hmmer/) 36 and Phyre2 (http://www.sbg.bio.ic.ac.uk/phyre2/html/page.cgi?xml:id=index) were used. The 3D structures for all investigated STT3 proteins were verified by both geometric and energetic measuring by the following servers: VERIFY3D to determine the compatibility of an atomic model (3D) with its sequence 37 and ERRAT to analyze the statistics of nonbonded interactions between different atom types 38; Tmscore (https://zhanglab.ccmb.med.umich.edu/TM-score/) to calculate RMSD. Protein models of open and close states of STT3 were generated through multitemplate comparative modeling.

## Results

### Identification and phylogenetic analyses of STT3 genes reveal independent duplication events in the plant and animal lineages

STT3 homologue genes are widespread across three domains of life (bacteria, archaea, and eukaryotes). It was previously hypothesized that STT3b in animals was similar to STT3a in plants because these isoforms had more comprehensive functions than the other STT3 isoform. Thus, to clarify the evolution of *STT3* genes in eukaryotes, we identified 77 *STT3* genes from species covering major evolutionary nodes of plants (21 genomes), animals (12), and fungi (10).

Generally, all investigated eukaryotic genomes possessed few copies of the *STT3* genes. In both plant and animal genomes, we frequently detected two copies of the *STT3* genes, while all investigated fungi only had one *STT3* gene. The obtained genes were used to construct an unrooted STT3 phylogeny (Table S1). The *STT3* genes identified in the eukaryotic genomes clustered into four major clades, corresponding to plant *STT3a*, plant *STT3b*, animal *STT3a*, and animal *STT3b* (Fig. S1). The sister relationship of the two animal STT3 clades and the two plant STT3 clades suggested that STT3 genes in animals and plants are more likely resulted from two independent gene duplication events, rather than inherited from the common eukaryotic ancestor. The *STT3* from the yeast genome clustered with animal STT3b, in accordance with a previous report 39, 40, suggesting an orthologous relationship between fungal STT3 and animal STT3b, and an ancient loss of the STT3a orthologue in fungi.

**Figure 1:**
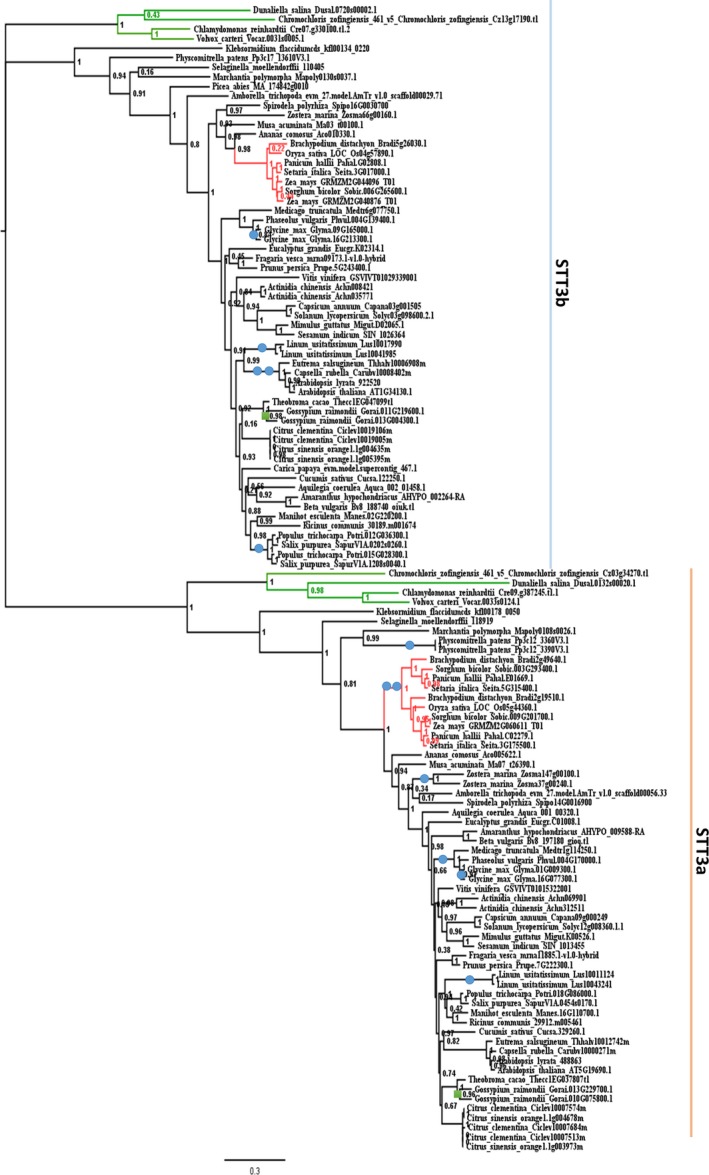
ML tree showing the evolutionary relationships among STT3 subtypes from land plants and algae. This ML tree was constructed based on an alignment of protein‐coding sequences. Numbers at nodes represent bootstrap support values, indicating whether the associated taxa clustered together in the bootstrap test (1000 replicates). Green branches indicate algae; pink branches indicate species of Phocaea; blue dots represent genome duplications; and green blocks indicate undefined polyploidization events.

Because the *A. thaliana stt3a* and *stt3b* mutants had different levels of salt sensitivity, we further added 48 plant genomes to our analysis to further explore the evolution of *STT3* genes in plants (Table S2). A phylogeny of plant *STT3* genes was reconstructed. *STT3* genes from plant genomes formed two distinct, well‐supported clades (Fig. 1). The presence of algal sequences in both clades suggested an ancient separation of *STT3* in the common ancestor of green plants. However, the ancient clades corresponding to STT3a and STT3b are conserved across the green plants, with most genomes surveyed contain only one gene from each clade. Lineage‐ or species‐specific gene duplications were observed in both clades albeit at low frequency (Fig. 1, labeled with blue dots and green blocks). The overall low STT3a and STT3b copy numbers in plant genomes suggested that functional restrictions might have led to the rapid loss of the redundant copies generated by rounds of genome duplications in land plants 41, 42. Interestingly, most species of grass family contained two copies of STT3a, and some species of Malpighiales contained two copies of STT3b. However, it remains unclear whether these additional copies of STT3 have specific functions in these species.

### Plant STT3a and STT3b genes are different in gene structure

**Figure 2:**
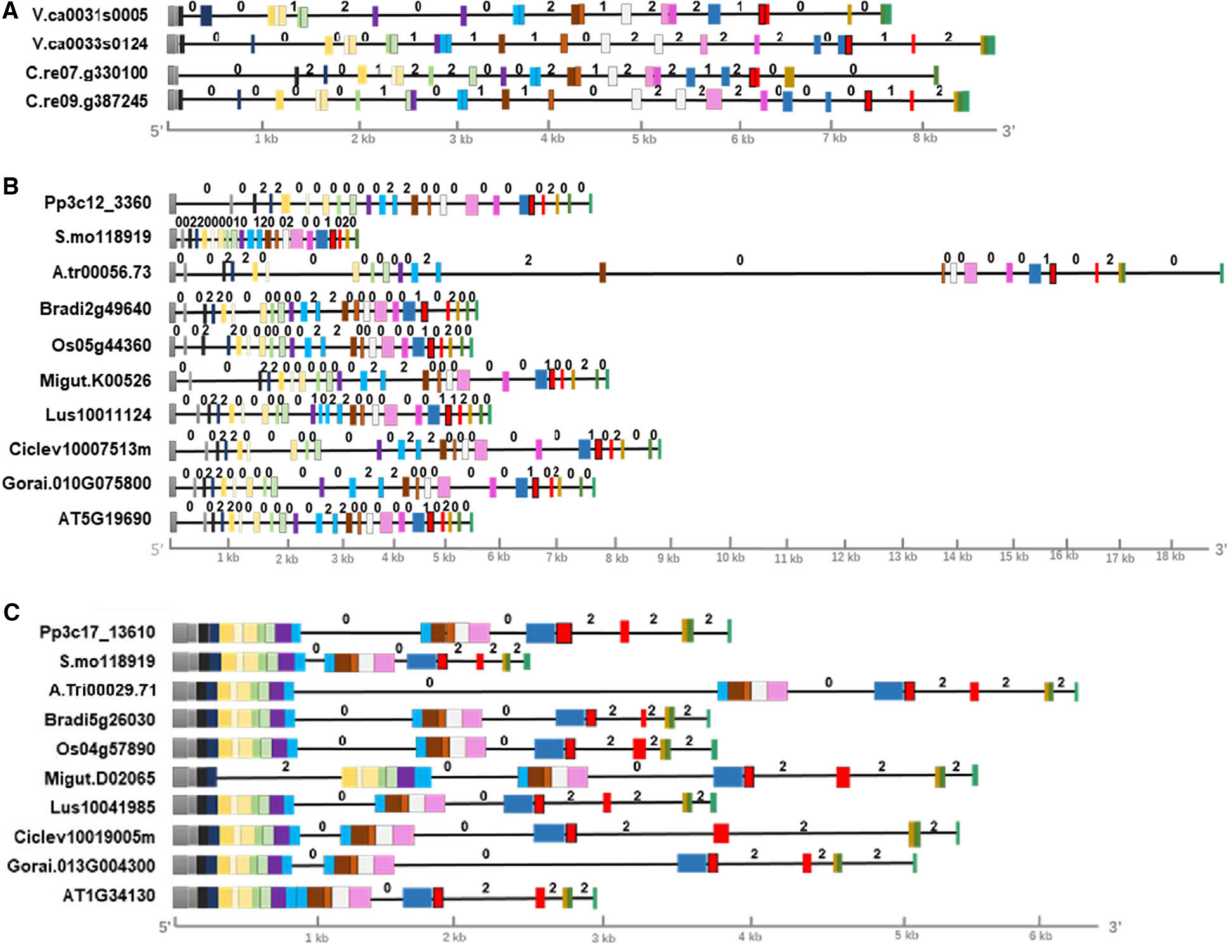
Schematic representation of *STT3* gene structure from translation start to stop sites in embryophytes and chlorophytes. (A) *STT3* gene structure in chlorophytes. The species names were listed as follows: *Volvox carteri *(*V.ca0033s0124*, *V.ca0031s0005*), *C. reinhardtii *(*C.re09.g387245*, *C.re07.g330100*)*.* (B) *STT3a* gene structure in embryophytes. (C) *STT3b* gene structure in embryophytes. Filled boxes indicate exons, and lines indicate introns. Exons are color‐coded based on sequence similarity with the corresponding exons on the *STT3* gene. Intron phases 0, 1, and 2 are marked above each intron. Exon–intron structures are shown to scale. The species were listed as follows: *Physcomitrella patens *(*Pp3c12_3360*, *Pp3c17_13610*), *Selaginella moellendorffii *(*S.mo118919*, *S.mo110405*), *Amborella trichopoda *(*A.Tri00056.33*,* A.Tri00029.71*), *Brachypodium distachyon *(*Bradi2g49640*, *Bradi5g26030*), *Oryza sativa *(*Os05g44360*,* Os04g5789*),* Mimulus guttatus *(*Migut.K00526*, *Migut.D02065*), *Linum usitatissimum *(*Lus10011124*,* Lus10041985*),* Citrus clementina *(*Ciclev10007513m*,* Ciclev10019005m*),* Gossypium raimondii* (*Gorai.010G075800*,* Gorai.013G004300*),* Arabidopsis thaliana *(*AT5G19690*, *At1g34130*).

Intron position and phase may evolutionarily conserved and are thus useful as additional indicators for evolution analysis of gene families 43. In animals, *STT3a* and *STT3b* had similar numbers of exons and similarly sized coding sequences. Short exons and long introns were usually scattered throughout both genes (Table S3). *STT3b* was typically much longer than *STT3a* in animals due to the tremendous variation in intron length. The structures of the *STT3a* and *STT3b* genes differed substantially between plants and animals. Although *STT3a* had similar numbers of exons in both plants and animals, *STT3b* had fewer exons in plants than in animals (Fig. 2B,C). Major differences were observed in the length of the sequences. The longest *STT3* was identified in *Amborella trichopoda*, while the shortest *STT3* was identified in *S. moeiiendorfii* (Table S4). These variations in gene length were primarily due to differences in the numbers and sizes of introns; this was consistent with the differences in cDNA sequences among species (Table S4). Most angiosperm *STT3a* genes had 23 exons, with the exception of *A. thaliana* (22) and *Linum usitatissimum* (24). In contrast, *STT3a* genes in mosses and gymnosperms had 22 exons each. This indicated an ancestral intron gain in angiosperm *STT3a*. All *STT3b* genes had six exons each, with the exception of *Mimulus guttatus* (7) and *A. thaliana* (5). These exceptions might be due to species‐specific intron gain and loss. Chlorophyta (e.g., *Chlamydomonas reinhardtii*) are obviously different from land plants, both *STT3* isoforms contained 14–16 exons (Fig. 2A). Variations were also observed in the lengths of the introns and exons. *STT3b* introns (318–4171 bp) were generally shorter than *STT3a* introns (1133–16 914 bp). Intron phases illustrate the position of the intron within a codon also differed between the two *STT3* types in plants. In *STT3a*, 72.7–76.2% of all introns were phase 0, while 22.7–23.8% were phase 2. In contrast, 25–33.3% of all *STT3b* introns were phase 0, while 60–75% were phase 2 (Fig. 2B,C). Furthermore, intron phases were highly conserved in each *STT3* type across land plants. No obvious variations in intron phase were identified between genes from different species in the same *STT3* clade. Our results suggested that both intron phase patterns and exon lengths are useful features for differentiating plant *STT3* isoforms. The conserved pattern of intron position and phase also provides simple features to distinguish plant STT3a and STT3b, as well as STT3s from animals.

### Promoter and expression analyses of the STT3 genes reveal different expression pattern

**Figure 3:**
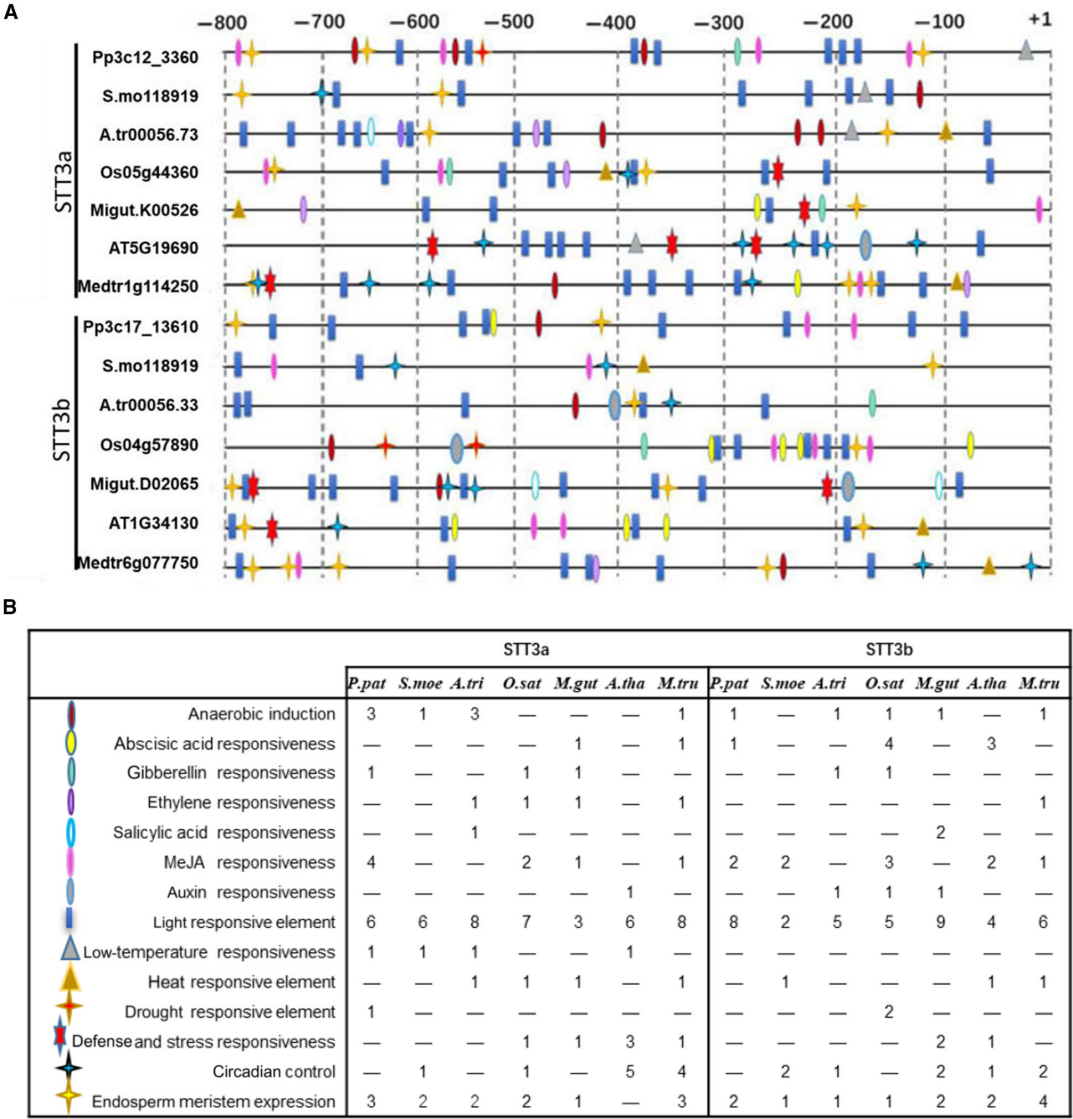
The regulatory elements identified in the 800‐bp region upstream of *STT3*. (A) Symbols correspond major regulatory elements were defined in (B). In (B), the frequency of each element in each representative species is given. The species were listed as follows: *Physcomitrella patens *(*Pp3c12_3360*, *Pp3c17_13610*), *Selaginella moellendorffii *(*S.mo118919*, *S.mo110405*), *Amborella trichopoda *(*A.Tri00056.33*, *A.Tri00029.71*), *Oryza sativa *(*Os05g44360*, *Os04g5789*), *Mimulus guttatus *(*Migut.K00526*, *Migut.D02065*), *Arabidopsis thaliana *(*AT5G19690*, *At1g34130*), *Medicago truncatula *(*Medtr1g114250*, *Medtr6g077750*).

In addition to gene structure, the promoters of the *STT3* genes might also affect their function by regulating gene expression. We used promoter analysis to identify the cis‐regulatory elements in the 800 bp upstream of the translation start site (ATG) of both *STT3* genes. These elements presumably respond to abiotic stressors, as well as to hormones (e.g., gibberellic acid and abscise acid). Regulatory elements associated with tissue‐specific expression (e.g., in the endosperm), and those with unknown function, were also identified (Fig. 3A; in both *STT3* genes, some regulatory elements, including TATA boxes, appeared frequently and thus are not shown in this diagram). The numbers of elements in the *STT3* gene promoter were counted and compared. The light‐response element was fairly well distributed across *STT3a* and *STT3b*. The anaerobic‐induction element was more commonly identified in STT3 genes from moss, gymnosperms, and basal angiosperms and might reflect adaptions to adverse circumstances. The low‐temperature response element was not identified in *STT3b,* indicating that responses to cold stress or freezing conditions might be mediated by *STT3a*. The ethylene‐response element was common in the *STT3a* genes of some angiosperm, but was absent in *STT3b* (Fig. 3B).

**Figure 4:**
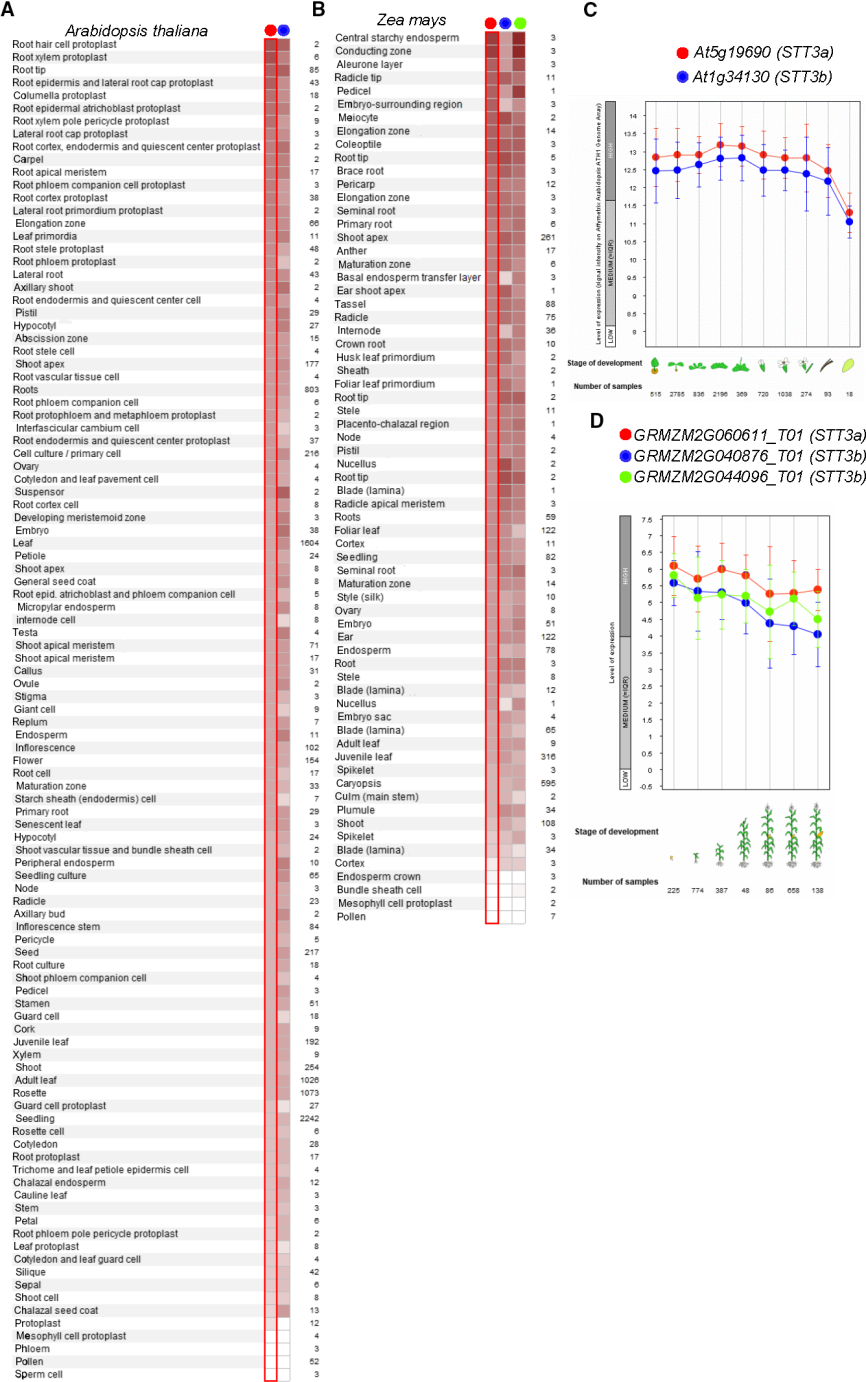
*STT3* gene expression in *Arabidopsis thaliana* and *Zea mays in silico*. (A, B) The relative gene expression of *STT3* in various tissues from (A) *A. thaliana* and (B) *Z. mays*. (C) *STT3* expression during the different developments of *A. thaliana* and (D) *Z. mays.* Error bars represent SEM.

An *in silico* expression analysis of *STT3* genes were performed. We extracted the expression data of *STT3a* and *STT3b* genes in each organism by Genevestigator software. *A. thaliana* and *Medicago truncatula* were chosen as representative dicots, while *Zea mays* (two copies of *STT3b*), *Oryza sativa* (one copy of *STT3a* and one copy of *STT3b*), and *Sorghum bicolor* (two copies of *STT3a*) were chosen as representative monocots. *STT3a* and *STT3b* gene expression levels were highest in the roots of all species (Figs 4A,C and S2A,D). *STT3a* was more highly expressed than *STT3b* in most tissues and developmental stages of *A. thaliana*, *Z. mays*, and *O. sativa,* even though *Z. mays* had two copies of *STT3b.* In contrast, *STT3b* was more highly expressed than *STT3a* in *M. truncatula* and *S. bicolor* (Figs 4B,D and S2B‐D). Therefore, *STT3* gene expression patterns might be species‐specific.

### Protein sequence divergence between STT3a and STT3b

**Figure 5:**
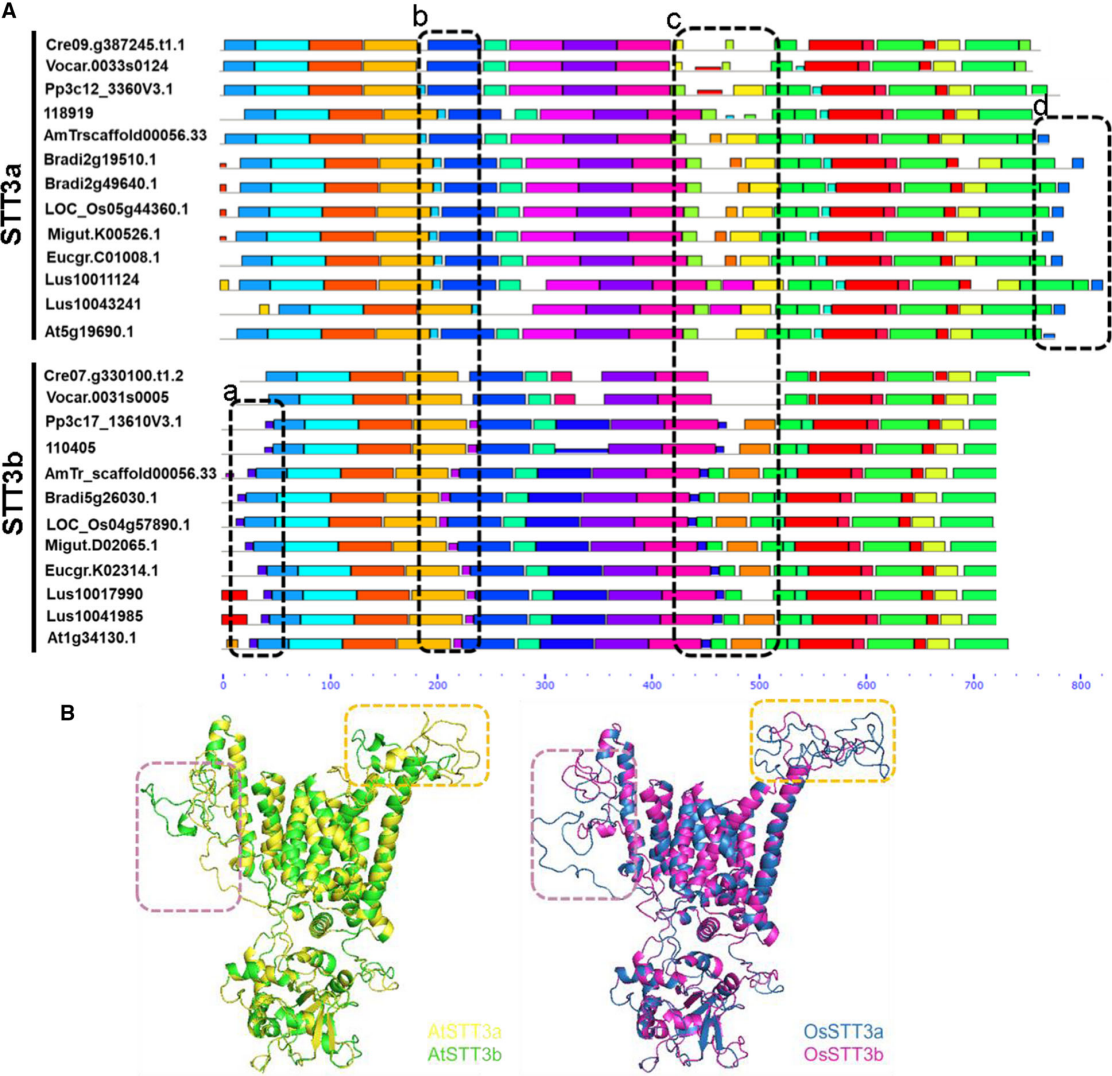
Motif similarities and predicted structures of plant STT3. (A) Protein sequence was compared using MEME to scan for 40 motif patterns in representative plant sequences. Black boxes indicate differences between STT3a and STT3b. (B) Predicted structures of the STT3 homologue in representative plants. The species and corresponding proteins are colored to match the predicted structures. Dotted boxes show differences between the motifs of STT3a homologue and STT3b homologue, based on the yeast Stt3p (PDB: http://www.rcsb.org/pdb/search/structidSearch.do?structureId=6ZEN) template.

To investigate the sequence divergence between STT3 proteins in a phylogenetic context, we used MEME website to compare protein motifs (Table S5). The parameters were set to detect as many as 40 motifs. With the exception of some motifs absent in algae, *STT3a* and *STT3b* shared almost all detected motifs with only four major differences (shown in dashed boxes in Fig. 5A). In all land plants, STT3b had motif 28, while STT3a did not (Fig. 5A, box a). STT3a and STT3b possessed motifs 25 and 29, respectively (Fig. 5A, box b). Angiosperm STT3a had a unique motif 26 at the C terminus (Fig. 5A, box d). The most divergent region was identified in the middle of both STT3 isoform sequences; in this region, STT3a contained motifs 20, 35, 36, and 19, while STT3b contained motifs 27, 21, and 18 (Fig. 5A, box c). These motifs were located in the central regions of the STT3a and STT3b protein sequences (Fig. 5A). When we aligned the STT3 proteins of *A. thaliana* and *O. sativa* (the representative dicot and monocot, respectively) using ClustalW, the sequence differences distributed across the central region corresponding to 433–510aa of AtSTT3a could be readily observed (Fig. S3).

Next, we generated 3D structures of STT3 isoforms from the representative dicot and monocot (*A. thaliana* and *O. sativa*) species using the 3D structure of yeast OST Stt3p (PDB ID: http://www.rcsb.org/pdb/search/structidSearch.do?structureId=6ZEN) as a template. Swiss‐Model and Phyre2 analyses indicated that yeast Stt3p had 50% sequence identity with *Arabidopsis* STT3. The quality factors estimated by ERRAT in conjunction with the graphical data recovered using Qmean 44 showed that energy values were negative which indicated a relatively stable energy environment. In addition, the TM score indicated that the RMSD was low (Table S7). These results suggested that the energy environment was favorable for the given amino acids.

The region encompassing motifs 18 and 19 forms divergent loop structure in the merged 3D model of STT3a and STT3b from both *A. thaliana* and *O. sativa* (Fig. 5B, yellow blocks), suggesting this region might influence the function of STT3a and STT3b. Another divergent region (Fig. 5B, pink box) was particularly interesting, and this area was near transmembrane (TM) helix 9 and included an extra loop 5 (EL5). We hypothesized that this region might change its conformation from helix to loose loop when binding to the substrate which is similar to the deformation of this part in archaea 45.

We next investigated whether this area would change differently in STT3a and STT3b in the catalytic process. Because no ligand binding state of STT3 has been identified in eukaryotes, we used AglB in open and closed states (PDB ID: http://www.rcsb.org/pdb/search/structidSearch.do?structureId=3WAK and PDB ID: http://www.rcsb.org/pdb/search/structidSearch.do?structureId=5GMY, respectively) as templates 28, 46. Although there is little similarity in the glycan structures transferred to acceptor protein between eukaryotes and prokaryotes, the OST catalytic domains are structurally and functionally related. These domains share a common topology, consisting of a multispan TM region and a C‐terminal globular domain located in the ER lumen of eukaryotes, and in the periplasm of bacteria 17, 27. Both PglB and AglB have crystal structures 27, 45, but pairwise distance analysis indicated that AglB was more similar to *A. thaliana* and *O. sativa* than PglB (Table S6). The black circler part in lower left of STT3a model showed a helix in apo state and a loop in peptide binding state, suggesting that STT3a might distort to improve peptide binding (Fig. S4). However, no similar conformational changes were identified in STT3b. Helix differences and characteristic motifs might be associated with the functional divergences between STT3a and STT3b.

### **Selection pressure analysis showed that central region had high** *K*_a_/*K*_s_ **values**

**Figure 6:**
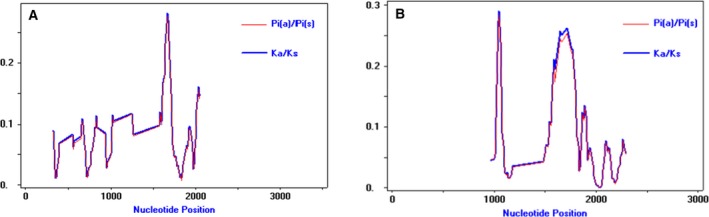
Rates of nonsynonymous and synonymous substitutions between STT3 orthologous protein‐coding sequences. The rate of nonsynonymous‐to‐synonymous substitution (*K*_a_/*K*_s_) in STT3 across 48 species. (A) STT3a and (B) STT3b were classified as shown in Table S2. Window length: 50 bp; step size: 10 bp.

Although our results indicated that STT3a and STT3b separated early in plant evolution and that these isoforms have both redundant and distinct functions, it remained unclear about the evolutionary history of the two isoforms. For example, one isoform might have been under strong constraint, while the other was under positive selection pressure to adapt or become vestigial. To analyze the selection pressures on different *STT3* isoforms, we calculated the rate of nonsynonymous‐to‐synonymous substitutions (*K*_a_/*K*_s_) in *STT3a* and *STT3b* of 48 protein‐coding sequences in plants (Table S2). Both *STT3* isoforms evolved under strong purifying selection, with a *K*_a_/*K*_s_ ratio of 0.075 for *STT3b* and a *K*_a_/*K*_s_ ratio of 0.081 for *STT3a*. As we previously showed that *STT3a* and *STT3b* shared 50% sequence identity, we hypothesized that selection pressure acted on only a small region of the *STT3* isoforms to influence their function. To test this, we used a sliding window *K*_a_/*K*_s_ analysis, with a setting window size of 50 bp and a step size of 10 bp. *K*_a_/*K*_s_ for *STT3b* peaked sharply near 1000 bp and (especially) at 1500 bp, but only one *K*_a_/*K*_s_ peak was observed in *STT3a* at ~ 1500 bp. This suggested that these regions have experienced more amino acid substitution than other regions (Fig. 6). The two *K*_a_/*K*_s_ peaks in STT3b generally coincided with differential structure in Fig. 5B (Fig. S3, pink and yellow boxes). Middle region (AtSTT3a: 433–510aa) were also identified near the second *K*_a_/*K*_s_ peak in *STT3b* (Fig. S3, green line). This suggested that these divergent regions were probably related to the functional differences of the two subunits.

## Discussion

### Ancient divergence offered inspiration of STT3s function in eukaryote

Both animals and plants have two different STT3 isoforms. Although it has been suggested that plant STT3a is similar to animal STT3b 18, our phylogenetic analyses suggested that independent gene duplication events generated the two STT3 isoforms in animals and plants. Fungal genomes had only one *STT3* gene, and these *STT3* genes formed a sister clade with animal *STT3b* genes. This suggested that the gene duplication that generated two copies of *STT3* in animals might have occurred before the separation of animals and fungi. This would imply that one copy of *STT3* was then lost in the fungi.

As early diverged animals were more likely to have multiple copies of *STT3b* and most fish possessed two *STT3a* genes and one *STT3b* gene, the two STT3 subtypes might have some different functions as well as some shared functions. As STT3b exhibits low oligosaccharide selectivity and high efficiency in mammals, it might suggest that STT3b transfers oligosaccharide chains to allow early diverged animals to adapt to complex environments, while STT3a might transfer oligosaccharides with lower efficiency in vertebrates. The two STT3a identified in fish might reflect adaptions to the multivariate aquatic environment, in contrast to the more stable terrestrial environment. Evidence for whole genomic duplication (WGDs) has been detected in all sequenced angiosperms, including at least five rounds of WGDs in *A. thaliana* 47, 48. Despite the large‐scale genome losses following these WGDs, most plants retain one copy of each STT3 subtype due to their important functions. As the grass family (except for *O. sativa*) possessed two *STT3a* genes and one *STT3b* gene, it was possible that an additional copy of STT3a remained after haploid meiosis to allow these species to tolerate certain stressors, including cambium deficiency and nutrient cotyledon. Thus, more STT3 copies might increase plant fitness by helping to balance growth and stress responses. This was consistent with previous studies, which showed that monocots possessed more gene families than dicots 49, 50. In contrast to duplicates created by WGDs, small‐scale duplications tend to be retained in some plant species like Malpighiales due to dosage‐balance constraints opposing their loss 51, 52. Thus, each of these duplications might have evolved different functions in separate plant lineages. Although many duplicates (paralogs) are lost after duplication, some undergo partial retention of ancestral functions (subfunctionalization) and the others are maintained after neofunctionalization 53, 54. Duplication patterns in individual gene families still require extensive investigation.

### Structures and gene expression differed between STT3 isoforms in plants

Introns and promoters both could regulate gene expression through different mechanisms. Introns may be considered as evolutionary fossils in a gene family, with intron position and phase serving as diagnostic tools with which to validate phylogenies 55, 56. Both *STT3a* and *STT3b* contained approximately the same number of exons, although *STT3b* genes were always longer than *STT3a* genes in animals. This was consistent with the greater efficiency and glycosylation ability of STT3b. The structures of plant *STT3a* and *STT3b* genes were similar to those of animal STT3 genes, but there were significant differences in intron length and intron number between *STT3a* and *STT3b* in plants. Based on the high similarity between genomic sequences, *STT3a* always had 23 exons, while *STT3b* typically had 6 exons. In contrast to land plants, algal *STT3a* and *STT3b* genes were of similar length and had similar numbers of introns numbers.

In *A. thaliana,* an average of 79% of the nuclear protein‐coding genes contains introns, and the average exon size is 250 bp 57. Because STT3 genes contained ~ 757 amino acids in average, the expected intron number was nine. Intron theory implies two possible scenarios. In the first scenario, *STT3a* acquired introns as suggested by intron‐gain theory. In the second scenario, *STT3b* lost introns as suggested by intron‐loss theory. *STT3a* may have evolved consistently with intron‐gain theory, and STT3a may thus have increased numbers of functions 58. This might indicate the massive loss and gain *STT3b* introns. In eukaryotes, both the number and the position of most introns reflect diverse histories of intron gains and losses 59, 60. Excess phase zero introns might indicate exon shuffling, as exon shuffling occurs frequently if introns are in the same phase 58. In addition to intron–exon structure, intron phase distinguished *STT3a* and *STT3b* in plants. The frequency of phase 0 introns in *STT3a* (72.7–76.2%) supported intron gain or duplication over evolutionary time. Present intron–exon patterns reflect past events and may inform evolutionary reconstructions. Tree and gene structure indicated that although plant STT3a potentially has similar functions to STT3b in animals, the evolutionary history and functional development of these isoforms are entirely different.

Despite differences in introns, STT3a and STT3b shared a series of TATA boxes and light‐response elements. However, various elements in the STT3 promoters led to isoform‐specific expression patterns in plants. This might lead to the isoform‐specific functions between STT3a and STT3b. Overall, anaerobic‐induction, low‐temperature‐response, and ethylene‐response elements were commonly found in the *STT3a* promoter. This might explain why *STT3a* was more highly expressed in most tissues and developmental stages of *A. thaliana* and *O. sativa*. That is, the upregulation of *STT3a* improved resistance to biotic and abiotic stressors.

### Characteristic motifs of STT3s and other OST subunits

Amino acid sequences may also reflect functional divergences. Motif comparisons indicated that motifs 18 and 19 were characteristic of STT3a and STT3b. When the PDB: http://www.rcsb.org/pdb/search/structidSearch.do?structureId=3WAK structure was used as a template, motif 18 formed a helix in STT3a, and motif 19 formed a free loop in the TM region of STT3b. The TM region might interact with other subunits, as this region was not in the C‐terminal containing the active center. When the PDB: http://www.rcsb.org/pdb/search/structidSearch.do?structureId=5GMY structure was used as a template, the peptide (324–345aa) encoded by *AtSTT3a* transformed from a helix to a loop like EL5 in AglB. The absence of this transformation in STT3b illustrated the difference in catalytic mechanisms between STT3a and STT3b. In addition to motif organization and expression patterns, the *K*_a_/*K*_s_ ratio also explains functional evolution. Although the two STT3 genes were under strong purifying selection, the regions with relatively high *K*_a_/*K*_s_ values included middle region of STT3. This implied that this region had evolved rapidly and that might related to the functional differences of the STT3a and STT3b.

Most proteins participate in interaction networks or act as subunits in protein complexes. The BioGRID (3.2.120) database shows that thousands of proteins interact physically with other proteins during various processes in yeast, *Arabidopsis* and humans 61, including DNA polymerases during replication 62 and ribosomes and proteasomes during protein synthesis and degradation 63, 64. OST is a heteromeric complex in yeast, suggesting that other subunits might help STT3 to transfer oligosaccharides. However, *AtSTT3a* and *AtSTT3b* did not rescue STT3 function in mutants with defective Stt3p 32. Cotransfection of AtSTT3a and AtSTT3b into yeast *stt3* mutants did not rescue growth in yeast lacking Stt3p (Fig. S5). This indicated that STT3 requires other subunits to function properly. It has been reported that the donor substrate recognized Wbp1p, the acceptor substrate recognized Ost1p, and the nascent translocated polypeptide might fit a groove by scanning for glycosylation sequences 65, 66. Mammalian ribophorin I affected the glycosylation of different peptides 67. A previous analysis demonstrated that Arabidopsis has two OST1 subtypes which interact with STT3a 21. Our evolutionary analysis of OST1 revealed that plant OST1 has diverged into two conservatively evolved clades in vascular plants (Fig. S6). The long‐term maintenance of the two OST1 clades suggests that plant STT3a/STT3b may interact with different OST1 subtypes to achieve distinct outputs. The deficiency of plant STT3s, separately or together, in rescuing yeast Stt3p mutant may due to the lack of a coevolved OST1 partner. To summarize, the differences we report here may underlie the functional divergence of plant STT3s.

## Conflict of interest

The authors declare no conflict of interest.

## Author contributions

GN designed the project, did all the analysis, and wrote the draft. ZS analyzed the phylogenic tree and revised the draft concerning the evolution. CL and QJ performed the experiments. TC made language modifications. ZH revised the manuscript.

## Supporting information


**Fig. S1** **.** Representative phylogenic analysis of *STT3 *genes in eukaryotes. This unrooted phylogeny of catalytic STT3 subunit homolog was reconstructed using 77 representative eukaryotic sequences. Bootstrap values from maximum likelihood analyses are given on basal and major nodes. Colors on circular margin represent the taxonomic classifications of the sequences.
**Fig. S2** **.** Expression of STT3 genes in three angiosperms. The relative expression of STT3 gene in different tissues of (A) *Oryza sativa*, (B) *Medicago truncatula *and (C) *Sorghum bicolor*. (D) STT3 expression at different development stages of *Oryza sativa *(left), *Medicago truncatula *(middle) and *Sorghum bicolor *(right). Error bars represent SEM.
**Fig. S3** **.** Sequence alignment of *Arabidopsis thaliana *and *Oryza sativa *STT3 genes. Residues similar in all sequences are marked with red in the alignment. The sequence corresponding to divergence motif in middle region (AtSTT3a433–510aa) were noted black dotted line frame. Different structure parts framed in Fig. 5B between STT3a homolog and STT3b homolog were showed in corresponding colours (pink and yellow dotted frame). The sequence corresponding to high *K*_a_/*K*_s_ value were annotated in full line (STT3a: blue, STT3b: green) along the sequence.
**Fig. S4** **.** Predicted tertiary structure is shown for AtSTT3 homolog in apo and ligand binding state. AtSTT3a (Pink) and AtSTT3b (Orange) were simulated on the basis of template AglB (PDB: http://www.rcsb.org/pdb/search/structidSearch.do?structureId=3WAK for apo‐state, PDB: http://www.rcsb.org/pdb/search/structidSearch.do?structureId=5GMY for peptide binding state). The part in black dotted frame were the proposed allosteric region between apo and peptide binding state. The a and c boxes are the regions containing EL5 that change from helix to free loop when STT3a goes from unbound to bound. Boxes b and d contain motif18 and 19 specific to STT3a and STT3b, respectively. In this region, both STT3a and STT3b have structural changes from unbound state to bound state.
**Fig. S5** **.** Neither AtSTT3a or AtSTT3b can rescue the yeast stt3 mutant. (A) Arabidopsis STT3s have incapacity in rescuing yeast STT3 mutant. WT (SS328) or yeast mutants (*stt3a‐4*) transformed with *YEp352 *(*vec*), *pSTT3*, *AtSTT3a *and *AtSTT3b *were cultured to mid‐log phase in liquid minimal medium lacking uracil. Serial 1:10 dilutions starting at 5 × 105 cells were spotted onto plates containing minimal medium lacking uracil. *Vec *is an empty vector YEp352 which serves as a negative control. p*STT3 *is yeast *STT3p *coding sequence in YEp352 which serves as a positive control. *AtSTT3a *and* AtSTT3b *were constructed on the basis of *pSTT3*. The Arabidopsis coding sequence were PCR amplified and digested with restriction enzymes, and ligated into the BamHI/NheI sites in the pSTT3 plasmid. So Arabidopsis cDNA were under control of yeast promoter. Plates were incubated at the labeled temperature for 3 days and then photographed. (B) Immunoblot analysis of degree of glycosylation of substrate protein. The transformants in A were grown at 23°C in minimal medium lacking uracil to midlog phase, shifted to 37° C, diluted after 3 h to an OD600 of 1.0. Cell extracts were prepared and used for CPY‐specific immunoprecipitation by 10% SDS/PAGE. CPY is the protein marker of yeast glycosylation. Except for yeast Stt3p, the STT3 protein of *Arabidopsis thaliana *could not restore its glycosylation level. The position of mature CPY and the di€fferent glycoforms lacking one to three N‐linked oligosaccharides (−1 to −3) are indicated.
**Fig. S6** **.** Schematic phylogenetic diagram of OST1 subunits. The unrooted phylogeny tree of the OST1s homolog was constructed using 106 representative eukaryote protein sequences by mega 5. Bootstrap values from maximum likelihood analyses are given on basal and major nodes. Colors on branch represent the taxonomic classifications of the sequences.
**Table S1** **.** 77 STT3 genes from diverse genomes of fungi, animals and plants.
**Table S2** **.** STT3 genes from diverse genomes in plants.
**Table S3** **.** STT3 gene structure and protein length comparison for representative species in animal.
**Table S4** **.** Comparison of length and identity of gene sequence for STT3 embryophyte and chlorophyte. All the sequences were compared to C.sub 40289.
**Table S6** **.** Pairwise distances calculation of STT3s in different species. The pairwise distances program in mega 5.0 was used to calculate genetic distance among these species amino acid sequences. Bootstrap was 500, model was poisson. A lower value indicates more lower genetic distance.
**Table S7** **.** Various average energy parameters of each system after Molecular Dynamics (MD) simulation analysis.

**Table S5** **.** The motif analysis details correspond to Fig. 5A. Pictogram is a sequence in every motif block, expressed in amino acid frequency. Width is the number of amino acids in motif.The colors of blocks correspond to the colors of motif in Fig. 5A.
