# Supplementary material for: Comparative and evolutionary analyses of the divergence of plant oligosaccharyltransferase STT3 isoforms
Source: FEBS Open Bio. 2020 Feb 19;10(3):468–83. doi: 10.1002/2211-5463.12804 (PMC7050244; doi:10.1002/2211-5463.12804)
Supplement: Supplementary file 1 — Fig. S1 . Representative phylogenic analysis of STT3 genes in eukaryotes. This unrooted phylogeny of catalytic STT3 subunit homolog was reconstructed using 77 representative eukaryotic sequences. Bootstrap values from maximum likelihood analyses are given on basal and major nodes. Colors on circular margin represent the taxonomic classifications of the sequences. Fig. S2 . Expression of STT3 genes in three angiosperms. The relative expression of STT3 gene in different tissues of (A) Oryza sativa, (B) Medicago truncatula and (C) Sorghum bicolor. (D) STT3 expression at different development stages of Oryza sativa (left), Medicago truncatula (middle) and Sorghum bicolor (right). Error bars represent SEM. Fig. S3 . Sequence alignment of Arabidopsis thaliana and Oryza sativa STT3 genes. Residues similar in all sequences are marked with red in the alignment. The sequence corresponding to divergence motif in middle region (AtSTT3a433–510aa) were noted black dotted line frame. Different structure parts framed in Fig. 5B between STT3a homolog and STT3b homolog were showed in corresponding colours (pink and yellow dotted frame). The sequence corresponding to high K a/K s value were annotated in full line (STT3a: blue, STT3b: green) along the sequence. Fig. S4 . Predicted tertiary structure is shown for AtSTT3 homolog in apo and ligand binding state. AtSTT3a (Pink) and AtSTT3b (Orange) were simulated on the basis of template AglB (PDB: http://www.rcsb.org/pdb/search/structidSearch.do?structureId=3WAK for apo‐state, PDB: http://www.rcsb.org/pdb/search/structidSearch.do?structureId=5GMY for peptide binding state). The part in black dotted frame were the proposed allosteric region between apo and peptide binding state. The a and c boxes are the regions containing EL5 that change from helix to free loop when STT3a goes from unbound to bound. Boxes b and d contain motif18 and 19 specific to STT3a and STT3b, respectively. In this region, both STT3a and STT3b have structural changes [file FEB4-10-468-s001.pdf]

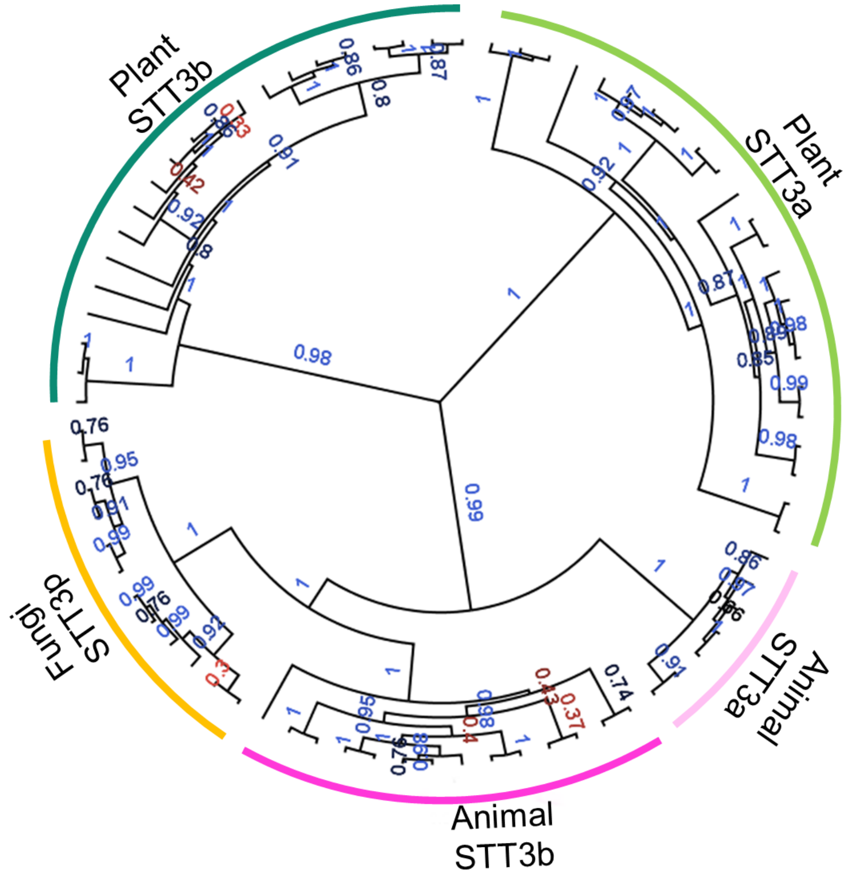

Supplemental Figure 1 Representative phylogenic analysis of *STT3* genes in eukaryotes. This unrooted phylogeny of catalytic STT3 subunit homolog was reconstructed using 77 representative eukaryotic sequences. Bootstrap values from maximum likelihood analyses are given on basal and major nodes. Colors on circular margin represent the taxonomic classifications of the sequences.

Kingdom	Species	Trascript name（locus name）	
		STT3a	STT3b
Fungi	*Anaeromyces sp*	296097|estExt_Genemark1.C_2640013	
	Coemansia reversa	41475|e_gw1.11.221.1	
	Conidiobolus coronatus	141291|CE52540_20412	
	Gonapodya prolifera	116611|estExt_Genewise1Plus.C_1080047	
	Lobosporangium transversale	322853|estExt_Genewise1.C_180315	
	Neurospora crassa	1315|NCU10497T0	
	Rhizophagus irregularis	32423|gm1.32311_g	
	Rhodosporidium toruloides	2262|RHTO_02269	
	Rozella allomycis	4167|O9G_002819	
	Saccharomyces cerevisiae	2547|YGL022W	
	Syncephalastrum racemosum	488864|fgenesh1_kg.3__1196__Locus2031v10rpkm1.92_PRE	
Animal	*Amphimedon queenslandica*	Aqu2.1.24984_001	Aqu2.1.36430_001
	Mnemiopsis leidyi	ML04031a-RA	ML04032a-RA
	Strongylocentrotus purpuratus	SPU_021968-tr	SPU_011178-tr<br>SPU_027777-tr
	Caenorhabditis elegans		T12A2.2.1
	Acyrthosiphon pisum<br>*ACYPI004183-RA*	ACYPI009548-RA	ACYPI004183-RA<br>ACYPI061915-RA
	Drosophila melanogaster	FBtr0345923	FBtr0084786
	Oryzias latipes	ENSORLT00000023172.1	ENSORLT00000002850.1<br>ENSORLT00000005368.1
	Xenopus tropicalis	ENSXETT00000038554.3	ENSXETT00000065166.1
	Mus musculus	ENSMUST00000120381.8	ENSMUST00000035010.9
	Homo sapiens	ENST00000392708.8	ENST00000295770.3
Plant	*Chlamydomonas reinhardtii*	Cre09.g387245.t1.1	Cre07.g330100.t1.2
	Chromochloris zofingiensis	_Cz03g34270.t1	Cz13g17190.t1
	Volvox carteri	Vocar.0033s0124	Vocar.0031s0005
	Physcomitrella patens	Pp3c12_3360V3.1<br>Pp3c12_3390V3.1	Pp3c17_13610V3.1
	Selaginella moellendorffii	118919	110405
	Amborella trichopoda	AmTr_v1.0_scaffold00056.33	AmTr_v1.0_scaffold00029.71
	Spirodela polyrhiza	Spipo14G0016900	Spipo16G0030700
	Brachypodium distachyon	Bradi2g19510.1<br>Bradi2g49640.1	Bradi5g26030.1
	Sorghum bicolor	Sobic.003G293400.1<br>Sobic.009G201700.1	Sobic.006G265600.1
	Oryza sativa	LOC_Os05g44360.1	LOC_Os04g57890.1
	Zea mays	GRMZM2G060611_T01	GRMZM2G040876_T01<br>GRMZM2G044096_T01
	Musa acuminata	Achr7T24990_001	Achr3T00140_001
	Mimulus guttatus	Migut.K00526.1	Migut.D02065.1
	Solanum lycopersicum	Solyc12g008360.1.1	Solyc03g098600.2.1
	Capsella rubella	Carubv10000271m	Carubv10008402m
	Arabidopsis thaliana	AT5G19690.1	At1g34130.1
	Phaseolus vulgaris	Phvul.004G170000.1	Phvul.004G139400.1
	Glycine max	Glyma.01G009300.1<br>Glyma.16G077300.1	Glyma.09G165000.1<br>Glyma.16G213300.1
	Medicago truncatula	Medtr1g114250.1	Medtr6g077750.1

Supplemental Table S1 77 STT3 genes from diverse genomes of fungi，animals and plants.

Phylum	Species	Trascript name（locus name）	
		STT3a	STT3b
Algae	*Dunaliellasalina*	Dusal.0132s00020.1	Dusal.0720s00002.1
	Volvox carteri	Vocar.0033s0124	Vocar.0031s0005
	Chlamydomonas reinhardtii	Cre09.g387245.t1.1	Cre07.g330100.t1.2
	Chromochloris zofingiensis	Cz03g34270.t1	Cz13g17190.t1
Moss Gymnosperm	*Physcomitrella patens*	Pp3c12_3360V3.1<br>Pp3c12_3390V3.1	Pp3c17_13610V3.1
	Marchantia polymorpha	Mapoly0108s0026.1	Mapoly0130s0037.1
	Selaginella moellendorffii	118919	110405
Basal angiosperm	*Amborella trichopoda*	AmTr_v1.0_scaffold00056.33	AmTr_v1.0_scaffold00029.71
Monocots	*Spirodela polyrhiza*	Spipo14G0016900	Spipo16G0030700
	Amaranthus hypochondriacus	AHYPO_009588-RA	AHYPO_002264-RA
	Zostera marina	Zosma147g00100.1<br>Zosma37g00240.1	Zosma66g00160.1
	Setaria italica	Seita.3G175500.1<br>Seita.5G315400.1	Seita.3G017000.1
	Panicum hallii	Pahal.C02279.1<br>Pahal.E01669.1	Pahal.G02808.1
	Sorghum bicolor	Sobic.003G293400.1<br>Sobic.009G201700.1	Sobic.006G265600.1
	Brachypodium distachyon	Bradi2g19510.1<br>Bradi2g49640.1	Bradi5g26030.1
	Zea mays	GRMZM2G060611_T01	GRMZM2G040876_T01<br>GRMZM2G044096_T01
	Ananas comosus	Aco005622.1	Aco010330.1
	Musa acuminata	Ma07_t26390.1	Ma03_t00100.1
	Oryza sativa	LOC_Os05g44360.1	LOC_Os04g57890.1
	Klebsormidium flaccidum	kfl00178_0050	kfl00134_0220
Dicots	*Mimulus guttatus*	Migut.K00526.1	Migut.D02065.1
	Aquilegia coerulea	Aquca_001_00320.1	Aquca_002_01458.1
	Gossypium raimondii	Gorai.010G075800.1<br>Gorai.013G229700.1	Gorai.011G219600.1<br>Gorai.013G004300.1
	Populus trichocarpa	Potri.018G086000.1	Potri.012G036300.1<br>Potri.015G028300.1
	Citrus clementina	Ciclev10007513m<br>Ciclev10007574m<br>Ciclev10007684m	Ciclev10019005m<br>Ciclev10019106m
	Citrus sinensis	orange1.1g003973m<br>orange1.1g004678m	orange1.1g004635m<br>orange1.1g005395m
	Manihot esculenta	Manes.16G110700.1	Manes.02G220200.1
	Capsicum annuum	Capana09g000249	Capana03g001505
	Cucumis sativus	Cucsa.329260.1	Cucsa.122250.1
	Ricinus communis	29912.m005461	30189.m001674
	Salix purpurea	SapurV1A.0454s0170.1	SapurV1A.0202s0260.1<br>SapurV1A.1208s0040.1
	Eutrema salsugineum	Thhalv10012742m	Thhalv10006908m
	Arabidopsis lyrata	488863	922520
	Arabidopsis thaliana	AT5G19690.1	At1g34130.1
	Beta vulgaris	Bv8_197180_gioq.t1	Bv8 188740_oiuk.t1
	Capsella rubella	Carubv10000271m	Carubv10008402m
	Eucalyptus grandis	Eucgr.C01008.1	Eucgr.K02314.1
	Actinidia chinensis	Achn069901<br>Achn312511	Achn008421<br>Achn035771
	Phaseolus vulgaris	Phvul.004G170000.1	Phvul.004G139400.1
	Medicago truncatula	Medtr1g114250.1	Medtr6g077750.1
	Fragaria vesca	mrna11885.1-v1.0-hybrid	mrna09173.1-v1.0-hybrid
	Glycine max	Glyma.01G009300.1<br>Glyma.16G077300.1	Glyma.09G165000.1<br>Glyma.16G213300.1
	Linum usitatissimum	Lus10011124<br>Lus10043241	Lus10017990<br>Lus10041985
	Vitis vinifera	GSVIVT01015322001	GSVIVT01029339001
	Prunus persica	Prupe.7G222300.1	Prupe.5G243400.1
	Sesamum indicum	SIN_1013455	SIN_1026364
	Solanum lycopersicum	Solyc12g008360.1.1	Solyc03g098600.2.1
	Theobroma cacao	Thecc1EG037807t1	Thecc1EG047099t1

Supplemental Table 2 STT3 genes from diverse genomes in plants.

Species	Exon		gene length（Kb）		CDS length（bp）		protein length（aa）	
	STT3a	STT3b	STT3a	STT3b	STT3a	STT3b	STT3a	STT3b
T.adhaerens	19	19	5.9	5.6	2078	2322	692	773
Fruitfly	6	5	2.9	7.2	2716	2885	713	774
C.savigniy	13/14	17/18	10.4	8.6	2547	2850	714	727
Zebrafish	16	16	41	92	2109	4224	702	805
G.gallus	19	15	8.3	61	2115	2337	705	779
M.musculus	18	16	46	81	2115	2472	705	823
H.sapiens	18	16	36	126	2115	2481	705	826

Supplemental Table 3 STT3 gene structure and protein length comparison for representative species in animal.

	Species	Gene		CDS		protein	
		Length(bp)	% Identity	Length(bp)	% Identity	Length(aa)	% Identity
STT3	C.sub40289	3948	—	2166	—	721	—
	V.car0033s0124	8773	34.4	2280	61.1	759	61.5
	V.car0031s0005	7564	38.6	2217	55.5	738	50.7
	C.rei07g330100	8134	47.2	2214	57.0	737	50.9
	C.rei09g387245	8510	34.2	2298	62.3	765	62.2
STT3a	P.pat123360	7754	44.1	2355	60.1	784	60.8
	S.moe118919	3443	42.5	2310	59.1	769	61.8
	A.Tri00056	19239	39.6	2325	58.6	774	61.0
	B.dis2g49640	5674	38.8	2421	58.7	806	59.5
	B.dis2g19510	6159	38.4	2382	58.4	793	61.1
	O.sat05g44360	5526	40.3	2364	59.6	787	61.1
	M.gut00526	8035	36.7	2337	59.2	778	60.4
	L.usi10011124	5886	41.2	2475	58.6	824	60.0
	L.usi10043241	6942	39.8	2370	58.9	789	59.5
	C.de10007513	9043	38.8	2349	59.3	782	60.1
	G.rai013G22970(	7040	36.7	2382	57.4	793	60.7
	G.rai010G07580(	7808	36.8	2367	58.2	788	59.9
	A.tha5G19690	5566	40.5	2340	59.0	779	60.1
	M.tru1g114250	8047	40.0	2322	58.6	773	59.4
STT3b	P.pat1713610	3991	39.9	2247	55.4	748	50.4
	S.moe110405	2550	36.6	2232	56.6	73	51.1
	A.Tri0002971	6372	37.4	2201	55.3	733	52.1
	B.dis5g26030	3813	39.9	2172	56.0	723	52.0
	O.sat04g57890	3772	39.1	2166	56.7	721	52.0
	M.gut02065	5653	36.9	2196	54.5	743	51.2
	L.usi10017990	3451	40.9	2229	55.4	742	51.3
	L.usi10041985	3844	40.2	2244	55.4	747	51.0
	C.de10019005	5552	38.7	2223	55.6	740	51.3
	G.rai011G21960(	4225	36.4	2163	54.8	720	52.1
	G.rai013G00430(	5197	37.9	2157	54.1	718	51.6
	A.tha1G34130	2986	40.6	2208	54.4	735	50.6
	M.tru6g077750	5788	35.7	2184	55.5	727	51.9

Supplemental Table 4 Comparison of length and identity of gene sequence for STT3 embryophyte and chlorophyte.
All the sequences were compared to C.sub 40289.

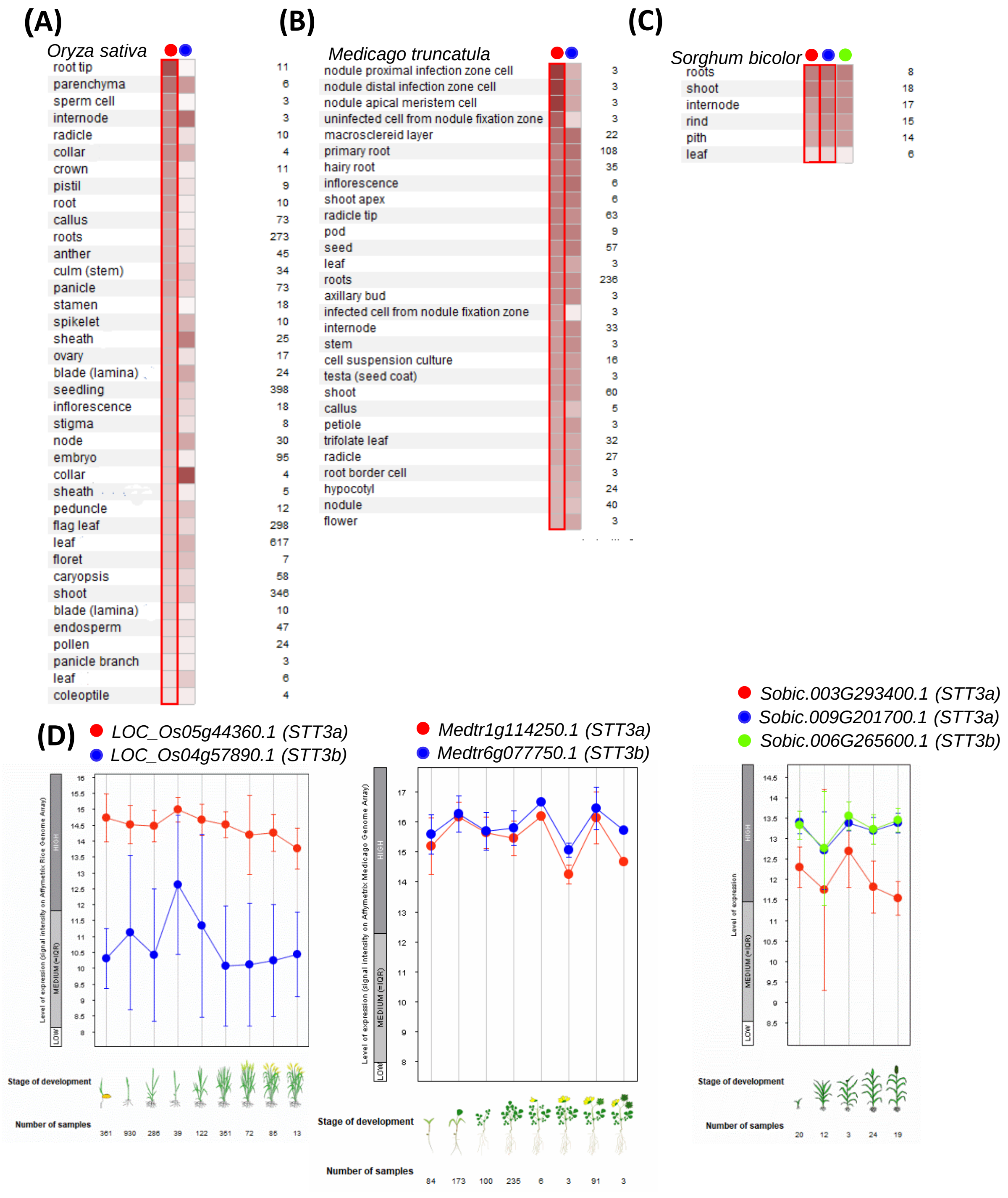

Supplemental Figure 2 Expression of STT3 genes in three angiosperms. The relative expression of STT3 gene in different tissues of (A) *Oryza sativa*, (B) *Medicago truncatula and* (C) *Sorghum bicolor*. (D) STT3 expression at differnent development stages of *Oryza sativa* (left), *Medicago truncatula* (middle) and *Sorghum bicolor* (right). Error bars represent SEM.

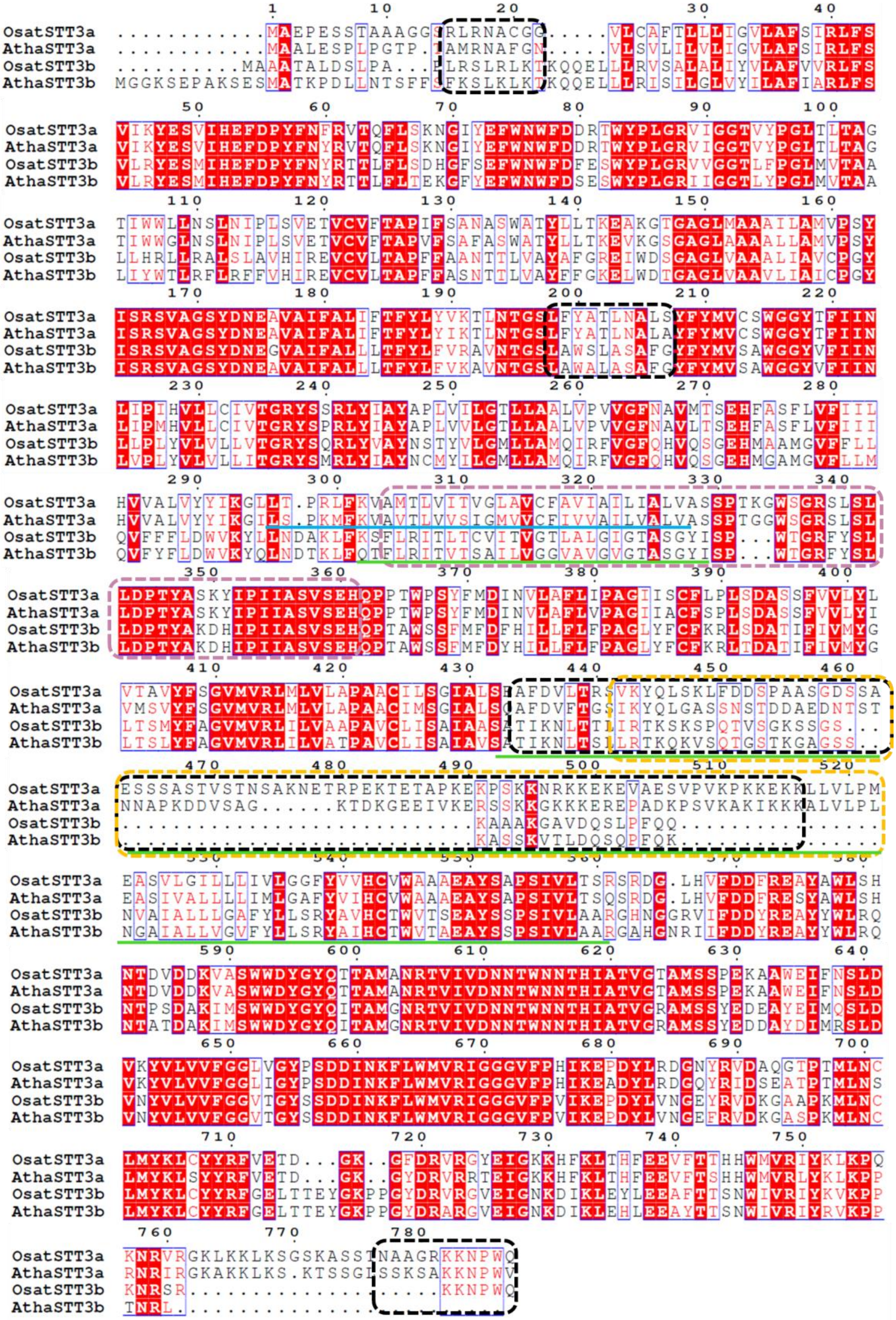
OsatSTT3a
AthaSTT3a
OsatSTT3b
AthaSTT3b

Supplemental Figure 3 Sequence alignment of *Arabidopsis thaliana* and *Oryza sativa* STT3 genes.

Residues similar in all sequences are marked with red in the alignment. The sequence corresponding to divergence motif in middle region (AtSTT3a 433-510aa) were noted black dotted line frame. Different structure parts framed in Figure 5B between STT3a homolog and STT3b homolog were showed in corresponding colours (pink and yellow dotted frame). The suquence corresponding to high Ka/Ks value were annotated in full line (STT3a: blue, STT3b: green) along the sequence.

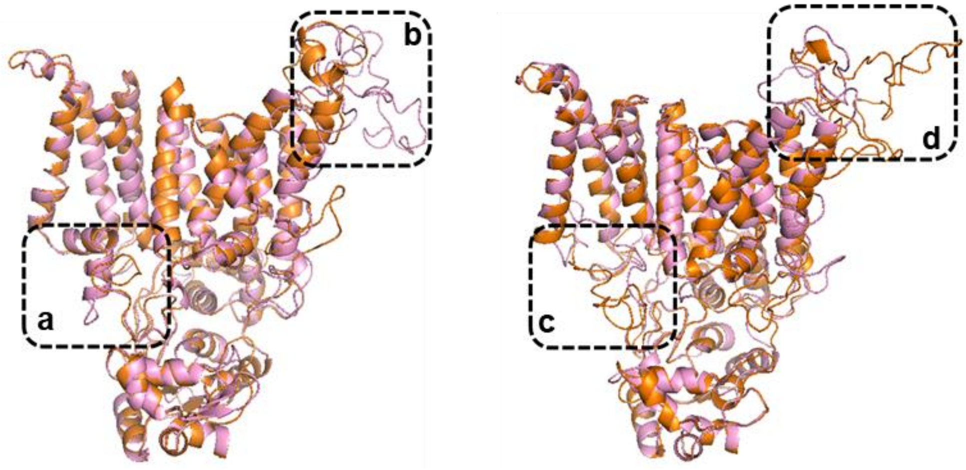

Apo-state Peptide binding state

Supplemental Figure 4 Predicted tertiary structure is shown for AtSTT3 homolog in apo and ligand binding state.

AtSTT3a (Pink) and AtSTT3b (Orange) were simulated on the basis of template AglB (PDB: 3WAK for apo-state, PDB: 5GMY for peptide binding state). The part in black dotted frame were the proposed allosteric region between apo and peptide binding state. The a and c boxes are the regions containing EL5 that change from helix to free loop when STT3a goes from unbound to bound. Boxes b and d contain motif18 and 19 specific to STT3a and STT3b, respectively. In this region, both STT3a and STT3b have structural changes from unbound state to bound state.

Pairwise distances	AtSTT3A	OsSTT3A	AtSTT3B	OsSTT3B	ScSTT3p	PglB	AglB
AtSTT3A		0.016	0.035	0.035	0.036	0.100	0.076
OsSTT3A	0.128		0.035	0.034	0.037	0.102	0.079
AtSTT3B	0.597	0.580		0.018	0.039	0.100	0.085
OsSTT3B	0.577	0.558	0.145		0.039	0.100	0.084
ScSTT3p	0.679	0.695	0.692	0.698		0.104	0.088
PglB	2.035	2.084	2.084	2.023	2.072		0.112
AglB	1.670	1.670	1.766	1.730	1.757	2.339	

Supplemental Table 6 Pairwise distances calculation of STT3s in different species. The pairwise distances program in Mega 5.0 was used to calculate genetic distance among these species amino acid sequences. Bootstrap was 500, model was poisson. A lower value indicates more lower genetic distance.

	structural validation statistics	*A.tha*			structural validation statistics	*A.tha*	
		STT3a	STT3b			STT3a	STT3b
	Seq Identity	19.17	18.78		Seq Identity	20.24	17.84
	Range	20 - 703	35 - 680		Range	20 - 703	35 - 680
	Coverage	0.74	0.79		Coverage	0.75	0.8
3WAK	GMQE	0.46	0.48	5GMY	GMQE	0.46	0.49
	QMEAN	-8.31	-7.92		QMEAN	-8.66	-8.06
	ERRAT	81.762	83.752		ERRAT	85.452	83.333
	TM-score	0.26	0.2339		TM-score	0.26	0.2916
	RMSD	3.74	3.96		RMSD	3.59	3.92
	structural validation statistics	*A.tha*			structural validation statistics	*O.sat*	
		STT3a	STT3b			STT3a	STT3b
	Seq Identity	50.45	49.63		Seq Identity	49.78	49.41
	Range	21-758	39-734		Range	22-764	26-725
	Coverage	0.87	0.91		Coverage	0.86	0.93
6EZN	GMQE	0.65	0.68	6EZN	GMQE	0.65	0.69
	QMEAN	-5.31	-4.99		QMEAN	-6.13	-4.57
	ERRAT	85.65	82.89		ERRAT	80.548	83.058

Supplemental Table 7 Various average energy parameters of each system after Molecular Dynamics (MD) simulation analysis.

A

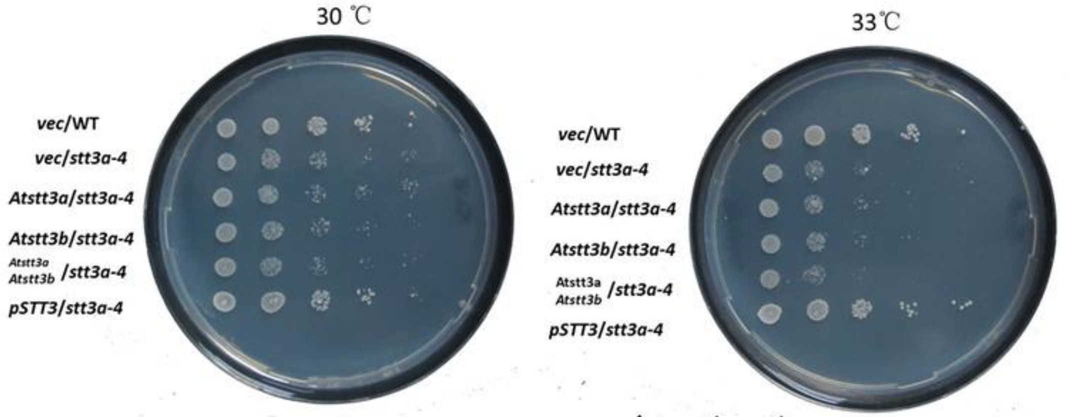

B

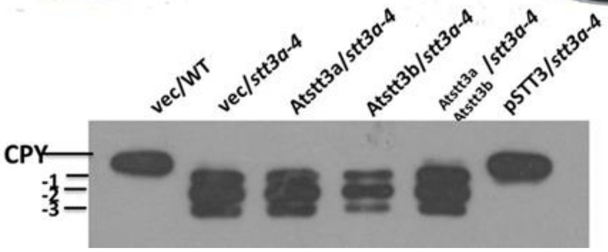

Supplemental Figure 5 Neither AtSTT3a or AtSTT3b can rescue the yeast stt3 mutant.

(A) Arabidopsis STT3s have incapacity in rescuing yeast STT3 mutant. WT (SS328) or yeast mutants (*stt3a-4*) transformed with *YEp352 (vec)*, *pSTT3*, *AtSTT3a* and *AtSTT3b* were cultured to mid-log phase in liquid minimal medium lacking uracil. Serial 1:10 dilutions starting at 5 $\times 10^5$ cells were spotted onto plates containing minimal medium lacking uracil. *Vec* is an empty vector YEp352 which serves as a negative control. p*STT3* is yeast *STT3p* coding sequence in YEp352 which serves as a positive control. *AtSTT3a and AtSTT3b* were constructed on the basis of *pSTT3*. The Arabidopsis coding sequence were PCR amplified and digested with restriction enzymes, and ligated into the BamHI/NheI sites in the pSTT3 plasmid. So Arabidopsis cDNA were under control of yeast promoter. Plates were incubated at the labeled temperature for 3 days and then photographed. (B) Immunoblot analysis of degree of glycosylation of substrate protein. The transformants in A were grown at 23°C in minimal medium lacking uracil to mid-log phase, shifted to 37° C, diluted after 3 h to an OD600 of 1.0. Cell extracts were prepared and used for CPY-specific immunoprecipitation by 10% SDS-PAGE. CPY is the protein marker of yeast glycosylation. Except for yeast Stt3p, the STT3 protein of *Arabidopsis thaliana* could not restore its glycosylation level. The position of mature CPY and the different glycoforms lacking one to three N-linked oligosaccharides (-1 to -3) are indicated.

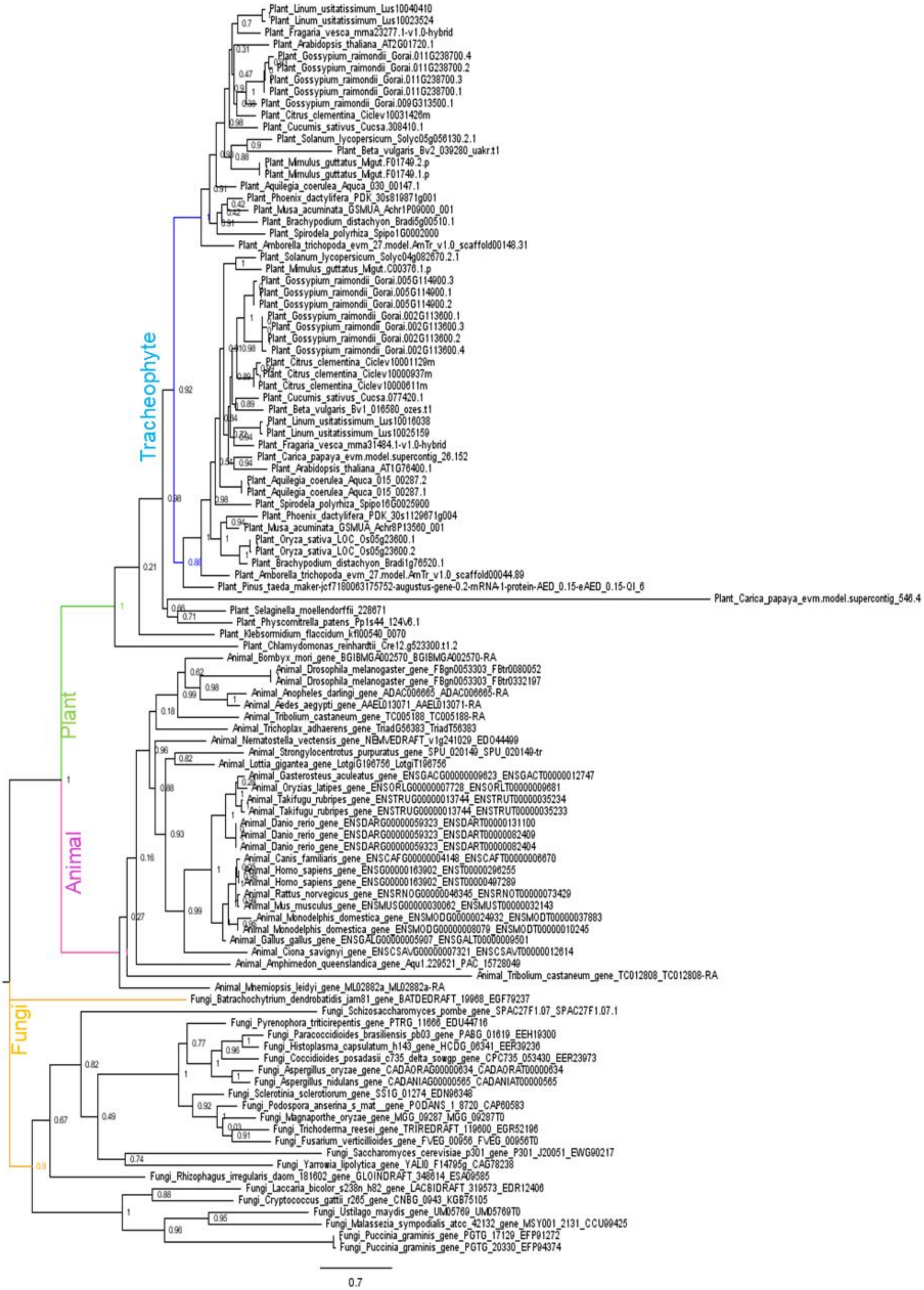

Supplemental Figure 6 Schematic phylogenetic diagram of OST1 subunits.

The unrooted phylogeny tree of the OST1s homolog was constructed using 106 representative eukaryote protein sequences by Mega 5. Bootstrap values from maximum likelihood analyses are given on basal and major nodes. Colors on branch represent the taxonomic classifications of the sequences.
