# Supplementary material for: Comparative and evolutionary analyses of the divergence of plant oligosaccharyltransferase STT3 isoforms
Source: FEBS Open Bio. 2020 Feb 19;10(3):468–83. doi: 10.1002/2211-5463.12804 (PMC7050244; doi:10.1002/2211-5463.12804)
Supplement: Supplementary file 2 — Table S5 . The motif analysis details correspond to Fig. 5A. Pictogram is a sequence in every motif block, expressed in amino acid frequency. Width is the number of amino acids in motif.The colors of blocks correspond to the colors of motif in Fig. 5A. [file FEB4-10-468-s002.pdf]

Motif	Pictogram	Width	Block	E-value
1	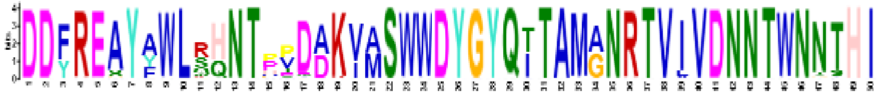	50		1.8e-1188
2	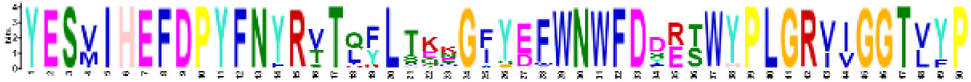	50		1.5e-3438
3	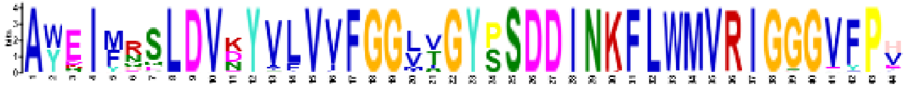	44		4.6e-895
4	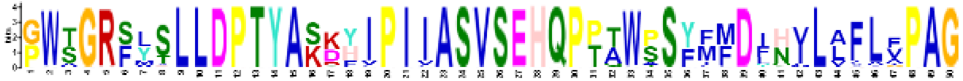	50		1.6e-982
5	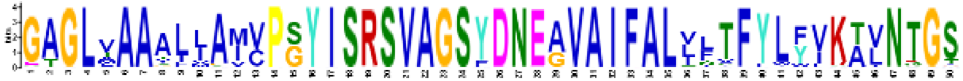	50		2.6e-883
6	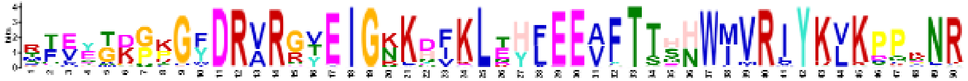	50		8.8e-876
7	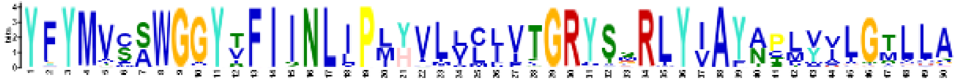	50		6.7e-822
8	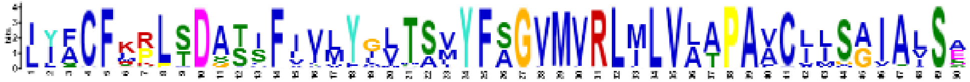	50		2.3e-781
9	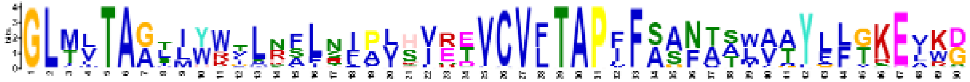	50		2.4e-690
10	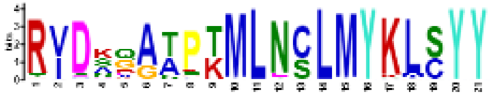	21		6.3e-382

11	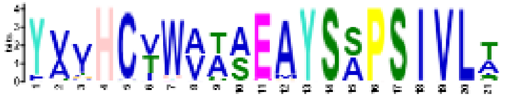	21		7.6e-352
12	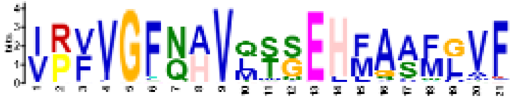	21		2.2e-296
13	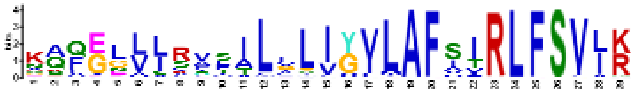	29		2.9e-321
14	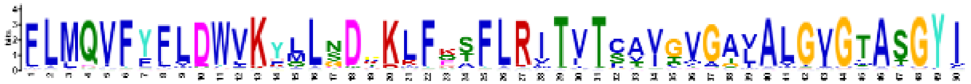	50		4.4e-202
15	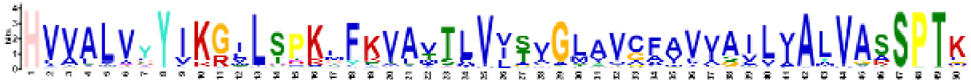	50		2.5e-204
16	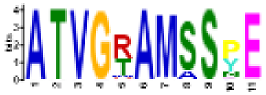	11		5.0e-170
17	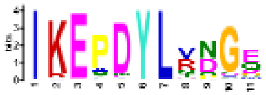	11		3.1e-154
18	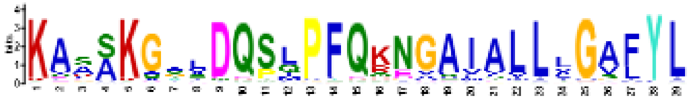	29		1.5e-098
19	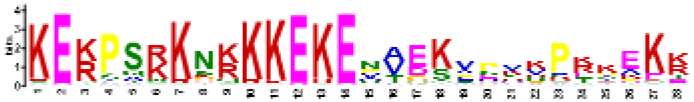	28		1.6e-095

20	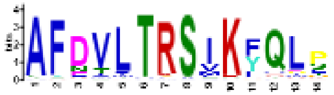	14		3.5e-052
21	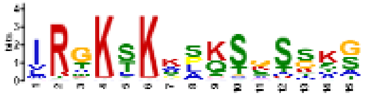	15		1.8e-048
22	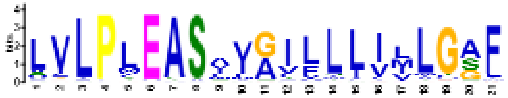	21		5.9e-050
23	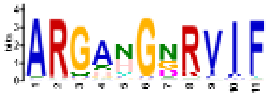	11		4.0e-036
24	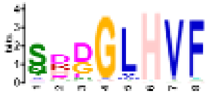	8		2.6e-833
25	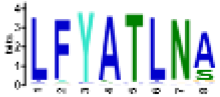	8		5.8e-025
26	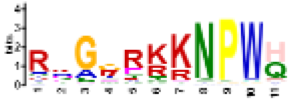	11		2.2e-020
27	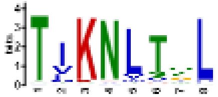	8		1.4e-010

28	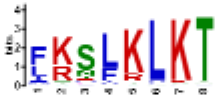	8	6.4e-008
29	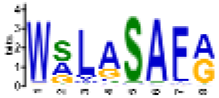	8	9.8e-007
30	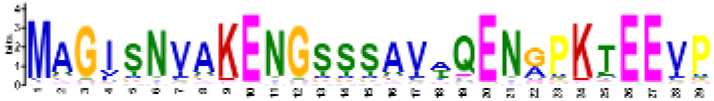	29	3.0e-006
31	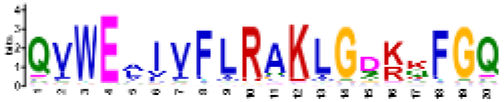	20	2.6e-001
32	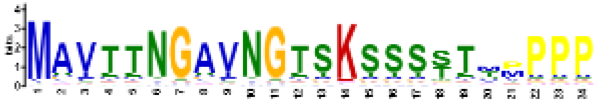	24	4.5e-001
33	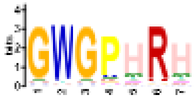	7	4.5e-001
34	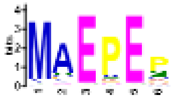	6	2.4e+003
35	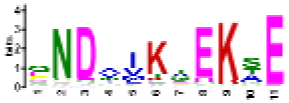	11	2.7e+003

36	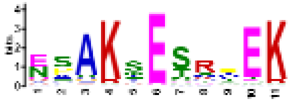	11		3.0e+003
37	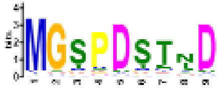	9		5.6e+002
38	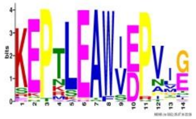	7		4.0e+003
39	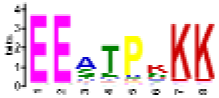	8		4.8e+003
40	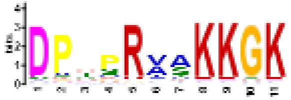	11		5.1e+003

Supplemental Table 5: The motif analysis details correspond to Fig. 5A.

Pictogram is a sequence in every motif block, expressed in amino acid frequency. Width is the number of amino acids in motif.The colors of blocks correspond to the colors of motif in Fig. 5A.
